# Biological evaluation of acellular bovine bone matrix treated with NaOH

**DOI:** 10.1007/s10856-022-06678-z

**Published:** 2022-07-15

**Authors:** Pengfei Li, Mengchun Feng, Xiantong Hu, Chunli Zhang, Jialiang Zhu, Gang Xu, Li Li, Yantao Zhao

**Affiliations:** 1 Institute of Orthopedics, Fourth Medical Center of the General Hospital of CPLA, 100048 Beijing, PR China; 2 Beijing Engineering Research Center of Orthopaedic Implants, 100048 Beijing, PR China; 3 Department of Orthopaedics, First Affiliated Hospital of Dalian Medical University, 116011 Dalian, PR China; 4 Key Laboratory of Molecular Mechanism for Repair and Remodeling of Orthopaedic Diseases, Dalian, 116011 Liaoning Province PR China; 5 State Key Laboratory of Military Stomatology, School of Stomatology, The Fourth Military Medical University, 710032 Xi’an, PR China

**Keywords:** Acellular bovine bone matrix, NaOH, Bone graft, Scaffold materials, Bone defect repair

## Abstract

We mainly proceed from the view of biological effect to study the acellular bovine bone matrix (ABBM) by the low concentration of hydrogen oxidation. After cleaning the bovine bone routinely, it was cleaned with different concentrations of NaOH and stained with hematoxylin-eosin (HE) to observe the effect of decellulization. The effect of bovine bone matrix treated with NaOH were observed by optical microscopy and scanning electron microscopy (SEM), and compared by DNA residue detection. Cell toxicity was also evaluated in MC3T3-E1 cells by CCK-8. For the in vitro osteogenesis detection, alkaline phosphatase (ALP) staining and alizarin red (AR) staining were performed in MC3T3-E1 cells. And the in vivo experiment, Micro CT, HE and Masson staining were used to observe whether the osteogenic effect of the materials treated with 1% NaOH solution was affected at 6 and 12 weeks. After the bovine bone was decellularized with different concentrations of NaOH solution, HE staining showed that ultrasonic cleaning with 1% NaOH solution for 30 min had the best effect of decellularization. The SEM showed that ABBM treated with 1% NaOH solution had few residual cells on the surface of the three-dimensional porous compared to ABBM treated with conventional chemical reagents. DNA residues and cytotoxicity of ABBM treated with 1% NaOH were both reduced. The results of ALP staining and AR staining showed that ABBM treated with 1% NaOH solution had no effect on the osteogenesis effect. The results of micro-CT, HE staining and Masson staining in animal experiments also showed that ABBM treated with 1% NaOH solution had no effect on the osteogenesis ability. The decellularization treatment of ABBM with the low concentration of NaOH can be more cost-effective, effectively remove the residual cellular components, without affecting the osteogenic ability. Our work may provide a novelty thought and a modified method to applicate the acellular bovine bone matrix clinically better.

**Figure:**
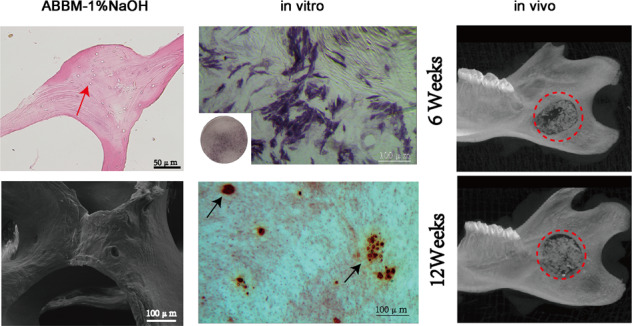
Graphical abstract

Graphical abstract

## Highlights

- We developed a low-cost and improved method of acellular bovine bone matrix with NaOH and the biological evaluation in vitro and in vivo was performed.
- Our work may provide a novelty thought and a modified method to applicate the acellular bovine bone matrix clinically better.

## Introduction

The global demand for bone replacements has been increasing [1], and many attempts have been made to develop suitable bone graft replacements for repairing bone defects. Tissue engineering constructs including additively manufactured pure silver antibacterial bone scaffolds [2] and highly permeable laser melted Ti6Al4V bone scaffolds [3] have been developed for bone defects. Besides, the use of bone graft for bone defect has become a clinically recognized bone reconstruction method [4]. Autogenous bone transplantation appeared in the form of cancellous bone, cortical bone and bone marrow transplantation. Since autologous transplantation is obtained from the patient, there are some risks [5, 6]. In the long-term study, fresh frozen allograft has been shown to be effective in the treatment of bone defects [7]. With the widespread use of graft techniques, such as the applications of drug delivery system [8–11], the demand for allogeneic bone also has increased dramatically. Due to the limited number of allografts [12], xenogeneic bone and composite materials have been successively developed and applied in the clinical practice [13]. In many cases, acellular scaffolds are constructed from animal bones and implanted in humans after the treatment [14]. However, the issues of cost, supply and risk of infection remain unresolved [15]. According to the rejection reactions involved in vascular events, they are classified as hyperacute, acute and chronic [16]. Acute rejection is caused by the action of one or more immune cells [17–20]. Therefore, the decellularization process is considered to be the key to the preservation of the extracellular matrix.

Based on the above, the scaffold materials used in surgery should be thoroughly acellular [21]. At present, detergents used commonly are sodium dodecyl sulfate (SDS), 3-[(3-cholinamide propyl) dimethylamino]-1-propanesulfonate (CHAPS), and Triton-X100/deoxycholate sodium (Triton/SDC) [22, 23]. Compared with SDS, CHAPS-BASED decellularization retained collagen and elastin, but the mechanical integrity of the scaffold was significantly reduced, while some elasticity loss occurred [22]. After the decellularization of goat ear cartilage with reagents (Na-deoxycholic acid + SDS and HCI + NaOH), the elastic modulus and hardness of scaffold materials were increased, and the structure of scaffold materials was also retained [24]. Acellular solutions based on NaOH have significant advantages in ionic inactivation of biomaterials [25, 26]. By comparing the acellular solution of NaOH with ordinary acellular solution, the acellular solution of NaOH is cost-effective and thus provides another potential detergent option for the future clinical application [27]. Thus, the low-cost and improved method of acellular bovine bone matrix for the clinical application is urged to be developed. Based on this, the main purpose of this study is to estimate the acellular bovine bone matrix (ABBM) by the low concentration of NaOH from the views of the residual cell components, the DNA residue and biological effect so as to provide a novelty thought and a modified method to applicate the acellular bovine bone matrix clinically better.

## Materials and methods

### Reagents and materials

Fresh cow femur (Institute of Orthopedics, Fourth Medical Center of the General Hospital of CPLA), Triton X-100 (chemical pure, national medicine group co., LTD.), hydrogen peroxide (30%, national medicine group co., LTD.), pepsin (purity ≥98%, national medicine group co., LTD.), alpha galactose enzyme (purity ≥99%, national medicine group co., LTD.), DNase I (1500 units/mg, Beijing Soleibao Technology Co., LTD).

### Preparation of acellular bone matrix

The soft tissue of fresh cow femur was removed and the bone cortex was excised by a high-speed cutting machine (automatic wheel slicing machine; Leica) to retain the cancellous bone, which was cut into cubes of 5 × 5 × 5 mm. Blood and bone marrow were cleaned by a water cannon. The samples were placed in a solution containing 1% triton X-100 and vibrated by ultrasound at 60 °C for 2 h, then residual triton solvent was rinsed thoroughly by warm water. The samples sequentially were cleaned with hydrogen peroxide and 75% alcohol for 2 h. The experimental group was treated with 0.5% NaOH, 1% NaOH and 3% NaOH solution in ultrasonic instrument for 30 min. Pepsinase, α galactoase and DNase I were used in turn. Before each treatment, it was fully cleaned with the distilled water. The materials were obtained after 24 h-freeze-drying in the vacuum freeze dryer.

### Evaluation of cleaning effect

Natural bovine bone matrix (NBBM), ABBM, and ABBM treated by NaOH (ABBM-NH) were decalcemized with 10% EDTA solution. About 4 weeks later, the decalcemized samples were put into an automatic dehydrator for dehydration, then transparentized, wax dipped, and paraffin embedded. The samples were sliced with a thickness of 6 μm, placed on a slide and baked at 60 °C for 30 min. Then, hematoxylin-eosin (HE) staining was performed.

Scanning electron microscope (SEM), the dried NBM, ABBM and ABBM-NH were fixed on the sample seat by the double-sided conductive adhesive and sputter-coated with gold. The microstructures of the scaffolds were observed using a scanning electron microscope.

### DNA residue detection

The tissue deoxyribonucleic acid content quantitative was detected by the kit (Animal tissue/cell genomic DNA Extraction Kit; Beijing Solai Bao science and Technology Co., LTD.) The tissue samples were weighed and freeze-dried, then were ground to powders and added in RNaseA solution, protease K and anhydrous ethanol, then centrifuged and the waste solution was discarded, then bleaching solution was added in. The cycle was repeated twice. At last, the scrubber was added to get the genomic DNA; Fluorescence was measured at 535 nm and DNA content was quantified by the standard curve.

### Cell toxicity detection

After the preparation of the materials, the samples were weighed and packed, and then irradiated (cobalt 60) for sterilization. The extracts were prepared with MEM medium containing 10% calf serum and samples at a volume ratio of 10:1. The suspension of mouse embryonic osteogenic precursor cells (MC3T3-E1) with the cell concentration of 2 × 10^4^/ml were divided into three groups and cultured in 5% CO_2_ incubator for 24 h. After the cells were attached, the extraction solution was added into the experimental group, and cultured for 1, 3, and 5 days respectively. The cytotoxicity was detected by CCK-8 kit (Beijing Prilai Gene Technology Co., LTD.). After 1 h reaction in 5% CO_2_ incubator, the absorbance was measured by a microplate analyzer at 450 nm wavelength. The calculated cytotoxicity formula was 100% × (OD_test_ − OD _background_)/(OD_control_ − OD_background_).

### Alkaline phosphatase (ALP) staining and quantification

The cell suspension was diluted to a concentration of 2 × 10^4^ cells/ml and inoculated in a 24-well plate with 1 ml/well. Each group was set up with three multiple wells and placed in the 37 °C and 5% CO_2_ incubator for 24 h. Osteogenic induction medium included MEM medium containing 10% serum, 10 mmol/l sodium β-glycerophosphine, 0.05 mmol/L vitamin C and 100 nmol/l dexamethasone; The experimental group was co-cultured with ABBM and ABBM-NH extracts, respectively. The complete medium containing osteogenic induction solution was replaced every 2–3 days, and the culture lasted for 14 days. The medium was gently washed with PBS for 3 times, fixed with 4% paraformaldehyde for 20 min, and gently washed with PBS for 3 times, 5 min each time. The staining was performed with BCIP/NBT kit and observed under a light microscope. ALP was quantified by ALP quantification kit (Beijing Pleilai Gene Technology Co., LTD.).

### Alizarin red (AR) staining and quantification

The cell suspension was diluted to a concentration of 2 × 10^4^ cells/ml and inoculated in a 24-well plate with 1 ml/well. Each group was set up with three multiple wells and placed in the 37 °C and 5% CO_2_ incubator for 24 h. The experimental group was co-cultured with ABBM and ABBM-NH extracts, respectively. The above osteogenic induction solution was replaced every 2–3 days, and the culture lasted for 21 days. The medium was gently washed with PBS for 3 times, fixed with 4% paraformaldehyde for 20 min, and gently washed with PBS for 3 times, 5 min each time. The staining was performed with AR solution and observed under a light microscope. Add 200 μl 10% cetylpyridine chloride to each well and it was placed for 30 min at room temperature. After mixed thoroughly, 100 μl was absorbed and moved to a 96-well culture plate, the absorbance was measured at 590 nm by microplate analyzer, and the degree of mineralization was analyzed semi-quantitatively.

### Animal experiment

Two-month-old male SD rats (180–220 g) were randomly divided into three groups with ten rats in each group. After feeding for 1 week, the operation was performed after the routine fasting and water prohibition. The rats were anesthetized with 3% sodium pentobarbital. After the effect of anesthesia, the rats were fixed on the operating table. The left mandible was peeled, and the surgical towel was spread. An oblique incision about 2–3 cm long was made along the lower edge of the mandible. Skin and subcutaneous tissue were cut layer by layer to separate muscles and expose the mandible body. A 5 mm diameter round defect was prepared with a mechanical drill, and normal saline was continuously added for cooling during the operation. ABBM and ABBM-NH scaffolds were placed in the mandibular defect, and no materials were placed in the blank control group. After the muscle and skin were sutured hierarchically, iodophor was used to disinfect the sutured area. Three days after the operation, 100,000 U penicillin sodium was intramuscularly injected continuously. The suture was removed 1 week after surgery, and the wound healing was observed. The rats were sacrificed at 6 weeks and 12 weeks, respectively.

### Micro-CT

The mandibles of sacrificed rats at 6 weeks and 12 weeks were removed and fixed with 4% paraformaldehyde for 24 h. Inveon MM CT (SIEMENS, Munich, Germany) was performed for the Micro-CT determination. Inveon Acquisition Workplace and Inveon Research Workplace (SIEMENS, Munich, Germany) were separately selected as the scanning and analyzing softwares. The resolution of the images was 9.21 μm and the scan condition including an X-ray tube potential of 80 kV, an X-ray intensity of 500 μA, and an exposure time of 400 ms. COBRA_Exxim (EXXIM Computing Corp., Livermore, CA) was selected as the CT reconstruction software.

### Histological evaluation

After the mandibles of sacrificed rats were fixed with 4% paraformaldehyde for 24 h, then the decalcification was performed with 10% EDTA solution, which was changed once a week, and the decalcification of the samples was examined after 4 weeks. Paraffin sections of the decalcified specimens were made and HE and Masson stainings were performed to evaluate the defect repair.

### Statistical analysis

All data in this study were statistically analyzed using SPSS (San Diego, USA). One-way analysis of variance was used to analyze the data. *P* < 0.05 was considered a significant difference.

## Results

### HE staining

**Fig. 1:**
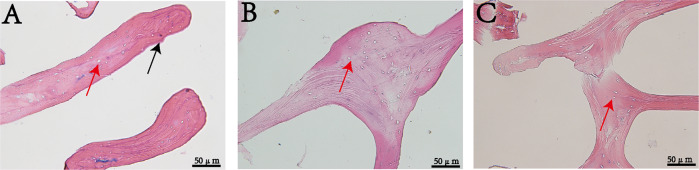
HE staining (*n* = 5). **A** 0.5% NaOH; (**B**) 1% NaOH; (**C**) 3% NaOH

HE staining results showed that some cells remained after treatment with 0.5% NaOH (Fig. 1A), and no obvious cell residues were found after treatment with 1 and 3% NaOH solution (Fig. 1B, C). However, the low concentration of NaOH could effectively remove the scrapie virus 263K [24], so 1% NaOH solution was used to treat scaffold materials in subsequent experiments.

### Effect of BBM treated with NaOH

**Fig. 2:**
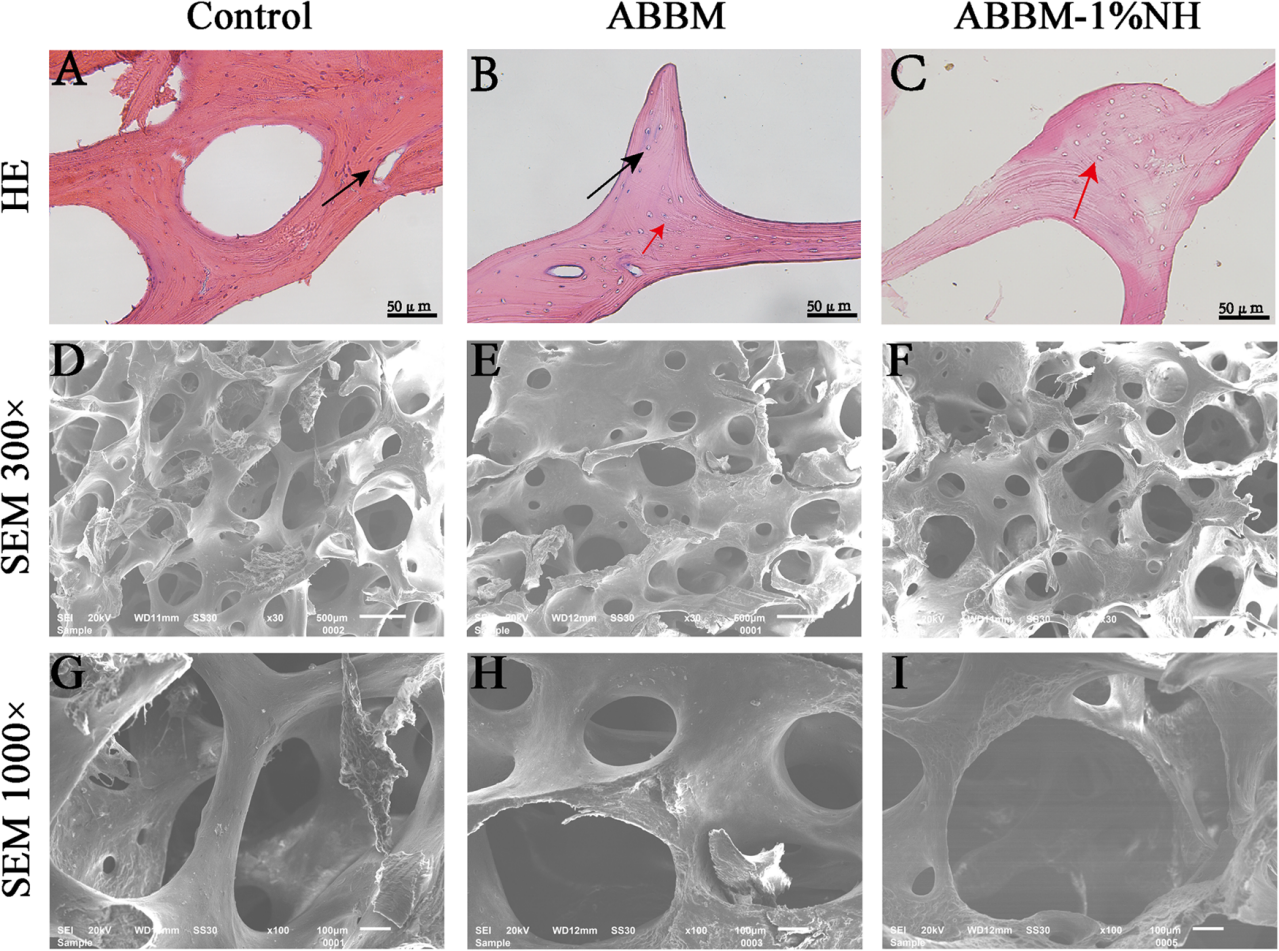
Effect of BBM treated with NaOH. HE staining (*n* = 5), (**A**) natural bovine bone matrix (NBBM); (**B**) acellular bovine bone matrix (ABBM); (**C**) ABBM treated by 1% NaOH (ABBM-1%NH). Scanning electron microscope (SEM) (*n* = 5), (**D**, **G**) NBBM; (**E**, **H**) ABBM; (**F**, **I**) ABBM-1%NH

HE staining results showed that there were a large number of cells in NBBM (Fig. 2A), some cell residues were observed in ABBM (Fig. 2B) treated with conventional chemical reagents. Compared with ABBM, no cell residues were observed in ABBM-NH (Fig. 2C). SEM observation showed that there were bone marrow and other impurities (Fig. 2D, G). SEM observation showed that ABBM-NH had less surface residual impurities than ABBM treated with conventional chemical reagents (Fig. 2E, F, H, I).

### DNA residue detection and CCK-8 testing

**Fig. 3:**
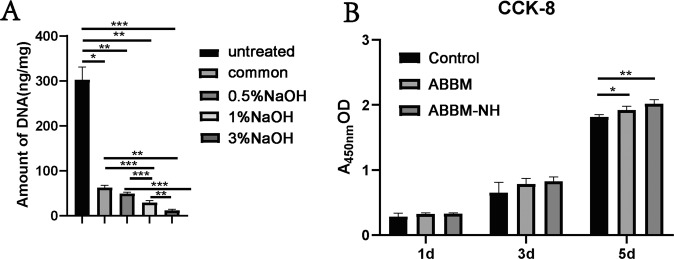
**A** DNA residue detection (*n* = 5); (**B**) CCK-8 testing (*n* = 5); *p < 0.05; **p < 0.01; ***p < 0.001

DNA residue detection of scaffold materials showed that the content of DNA in natural bovine bone matrix was significantly higher than that in other groups, while the content of DNA residue in ABBM-NH groups (1 and 3%) were significantly lower than that in ABBM (Fig. 3A).

Cytotoxicity results showed that the number of MC3T3-E1 cells increased with the prolongation of culture time after ABBM and ABBM-1% NH, which was detected by CCK-8 kit. The cells cultured with ABBM-NH and ABBM were superior to the control group on day 5 (*P* < 0.05), while there was no difference between the three groups on day 1 and day 3 (Fig. 3B).

### Detection of osteogenic activity in vitro

**Fig. 4:**
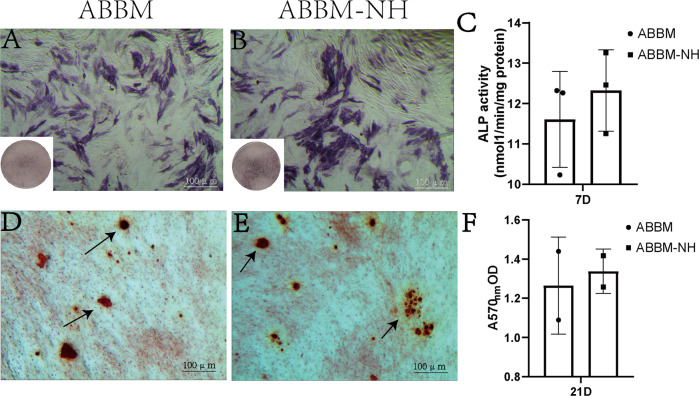
Detection of osteogenic activity in vitro. ALP staining (*n* = 5), (**A**) ABBM; (**B**) ABBM-NH; (**C**) ALP activity tested by BCIP/NBT kit. AR staining (*n* = 5), (**D**) ABBM; (**E**) ABBM-NH; (**F**) AR activity tested by microplate analyzer at 590 nm

The results showed that the activity of ALP in ABBM-NH was significantly higher than that in ABBM (*P* < 0.05), and there was no difference between ABBM-NH and ABBM (Fig. 4A–C). AR staining results showed that compared with ABBM (Fig. 4D–F), ABBM-NH had a larger positive area of differentiated calcium nodules, and there was no significant difference between the two groups (Fig. 4F) combined with the ALP results (Fig. 4C).

### Micro-CT

**Fig. 5:**
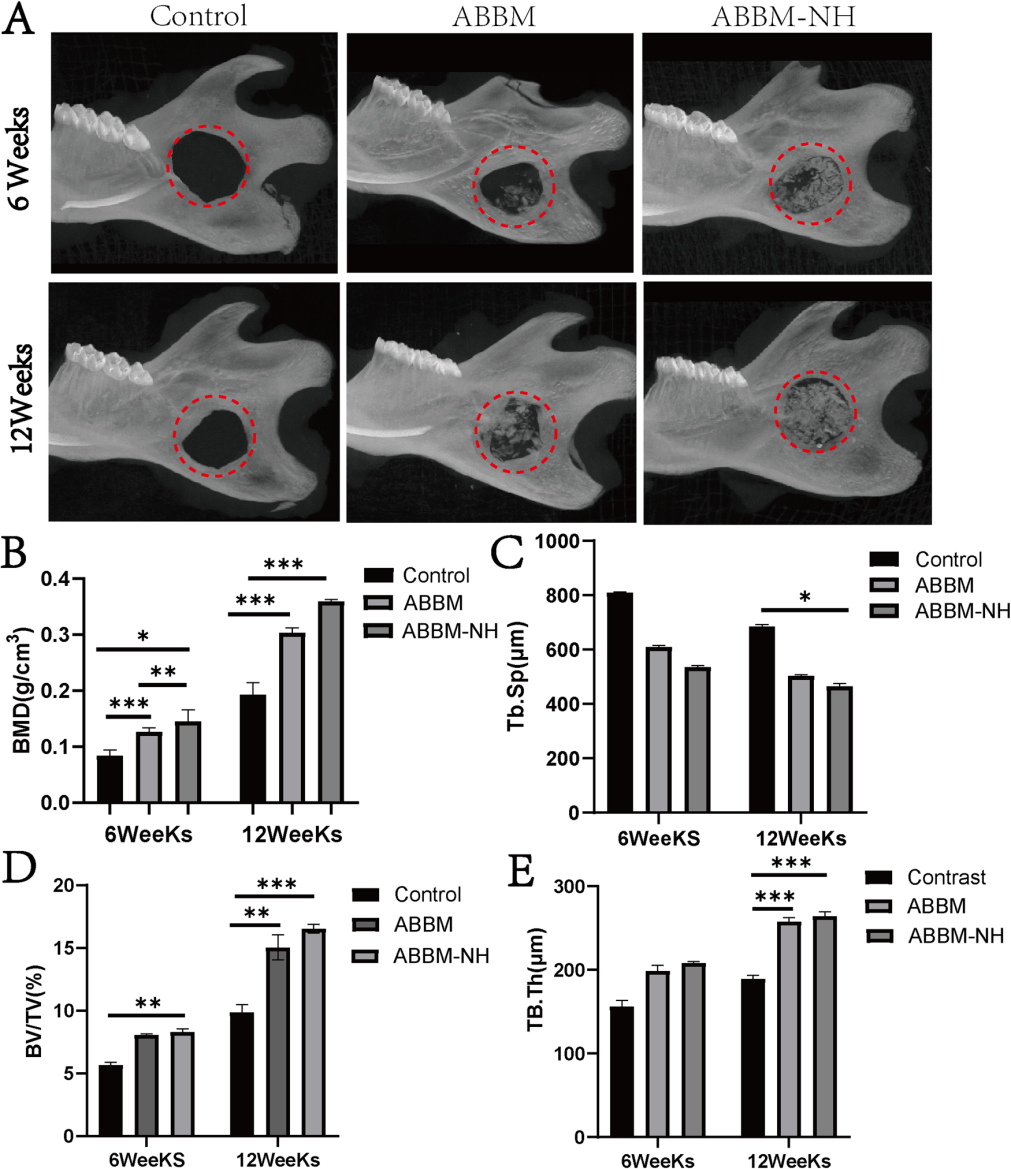
Micro-CT scanning of 6 and 12 weeks. **A** Micro-CT observation, *n* = 5; (**B**) bone mineral density (BMD), *n* = 5; (**C**) Tb.Sp, *n* = 5; (**D**) The bone mass/total volume (BV/TV), *n* = 5; (**E**) TB.Th, *n* = 5; *p < 0.05; **p < 0.01; ***p < 0.001

The results showed that scaffolds of ABBM and ABBM-NH at 6 weeks and 12 weeks were dispersed in the bone defect area (Fig. 5A). With the passage of time, scaffold materials would degrade to varying degrees. New bone volume percentage (%) and bone mineral density (BMD, g/cm3) were calculated by micro-CT software for quantitative analysis of newly formed bone tissue (Fig. 5B) (*P* < 0.05). At 6 weeks and 12 weeks, compared with the control group, bone mineral density and new bone volume ratio were significantly increased, trabecular bone thickness and trabecular space in ABBM and ABBM-NH were better than the control group (Fig. 5C–E).

### Histological observation

**Fig. 6:**
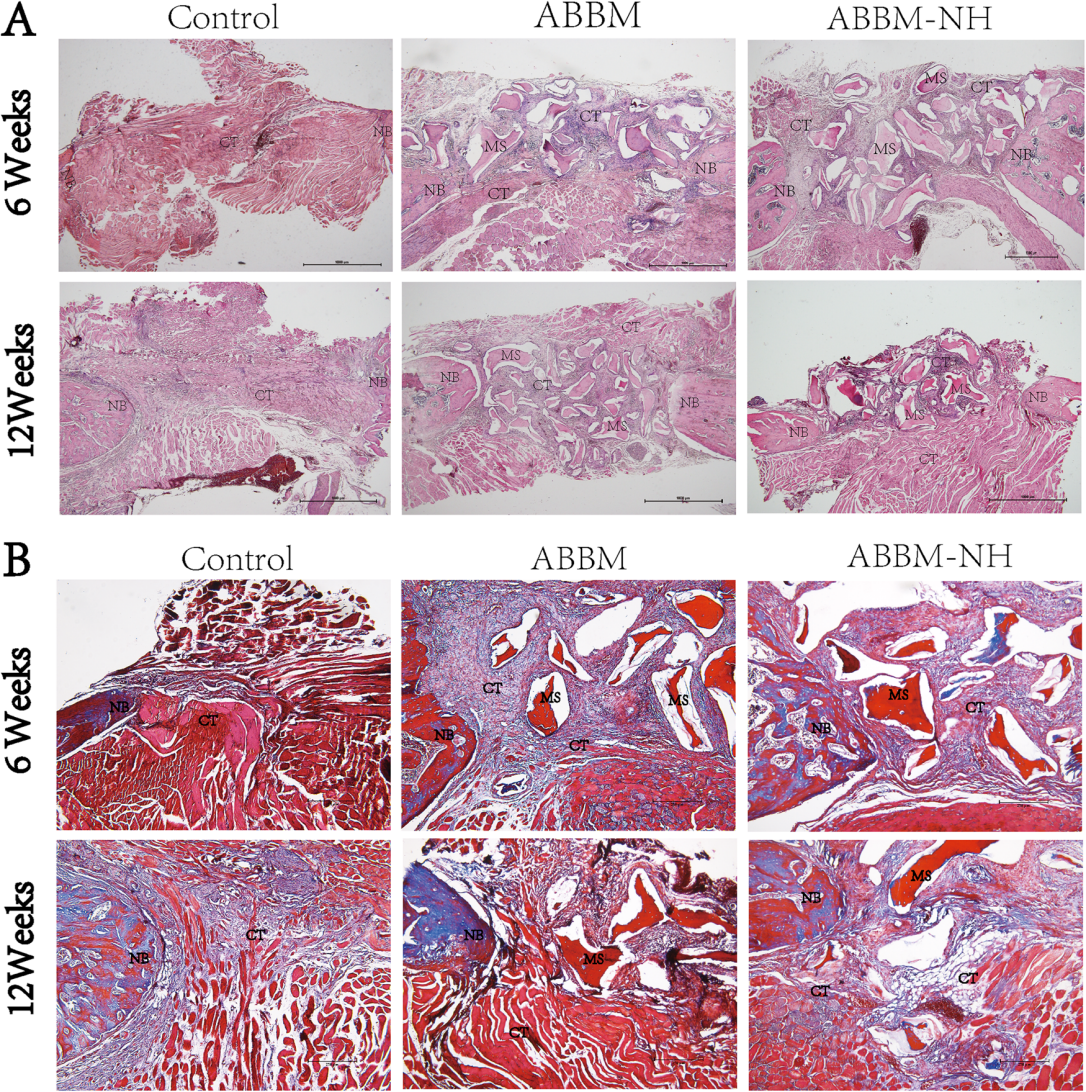
The histology observed at 6 weeks and 12 weeks after the operation. **A** HE staining (*n* = 5); (**B**) Masson staining (*n* = 5)

The results showed that the defect was filled with a large amount of fibrous tissue at 6 weeks after surgery. Compared with the growth at 6 weeks, there was part of new bone ingrowth at the edge of the bone defect at 12 weeks, while a small amount of new bone formed at 6 weeks. At 12 weeks, the materials in ABBM and ABBM-NH were partially degraded, and few materials remained (Fig. 6A, B).

## Discussion

In this study, the low-concentration NaOH solution was used to improve the acellular effect of ABBM. The acellular effects of different concentrations of NaOH solution showed that some cells remained in ABBM after treatment with 0.5% NaOH solution, while the acellular effects of both 1 and 3% NaOH solutions were significant. However, the low concentration of NaOH can effectively treat the infectivity of the scrapie 263K [28], so 1% NaOH solution was used to treat ABBM in the experiment.

The results of HE staining and SEM showed that 1% NaOH solution effectively removed the remaining cells treated with the conventional reagent. Cells cultured with the material leaching solution, according to the results, on the first day and the third day, it had no obvious difference between groups (Fig. 3B), On the 5th day, cell growth conditions of ABBM and ABBM-NH were obviously better than the control group, demonstrating decellularization based on NaOH solution in biological materials had significant advantages in the respect of ion inactivation as references [25, 26] reported. The DNA residue results (Fig. 3A) showed that the DNA residue in ABBM-NH was significantly lower than that in ABBM, indicating that 1% NaOH solution removed the remaining cells and reduced the DNA residue, which was consistent with the reported results [27]. ALP and AR stainings and quantifications (Fig. 4) showed that there was no significant difference in ALP staining and quantification between ABBM and ABBM-NH, and the results of AR staining and quantification also matched those of ALP staining and quantification. The acellular treatment with 1% NaOH solution did not affect the osteogenic activity of ABBM in vitro.

All the rats survived and were included for observation and analysis. There was no postoperative inflammation or infection at the surgical site. A three-dimensional image of the hard tissue of the rat mandible defect was shown in Fig. 5. In the in vivo experiment, micro-CT results showed that a small amount of new bone was generated at the defect edge of the ABBM and ABBM-NH at 6 weeks after implantation (Fig. 5). HE and Masson staining were used to observe bone regeneration in the rat mandibular defect model and showed soft tissue grew around the material (Fig. 6). Micro-CT results at 12 weeks showed the partial material degraded and new bone formed (Fig. 5A), keeping in line with the HE staining result.

At present, it is necessary to reduce the immunogenic response and reduce the cost of ABBM in clinical application. Acellular method based on NaOH was similar to other reagents, in general, under study, 1% NaOH solution is likely to be better in terms of cost and safety among the acellular solutions. There are similar methods of decellularization, for example, using ammonium hydroxide with Tryton X as a high pH detergent has been successful for the decellularization of a human-sized liver [29], and high pH NaOH-PBS solution without using detergents has been used for the decellularization of a rat lung [27]. At present, we did not compare the acellular effects of these similar methods of decellularization on the bone matrix. The presented methodology may be modified and updated for a better result in the near future. Another limitation of this study was that we did not observe the above results in nonrodent animals like a dog or a sheep. Therefore, further research should be conducted in nonrodent animals.

From the results of the present study, we can conclude that 1% NaOH solution effectively removed the cells remaining after conventional reagent treatment. In terms of removing extracellular matrix and eliminating DNA effectively, the acellular effect of NaOH solution is better than that of conventional acellular solution. The low concentration of NaOH acellular solution found by our group can be more cost-effective, effectively remove residual cells and reduce the occurrence of immunogenicity. Our work may provide a novelty thought to applicate the acellular bovine bone matrix clinically better.

## Conclusion

The acellular treatment of acellular bone matrix treated with the low concentration of NaOH can be more cost-effective, effectively remove the residual DNA components and reduce the immunogenicity without effect on the osteogenic ability of acellular bone matrix. Our work may provide a novelty thought and a modified method to applicate the acellular bovine bone matrix clinically better.
